# Isolation, speciation and antifungal susceptibility testing of *Candida* isolates from various clinical specimens at a tertiary care hospital, Nepal

**DOI:** 10.1186/s13104-017-2547-3

**Published:** 2017-06-24

**Authors:** Sundar Khadka, Jeevan Bahadur Sherchand, Bharat Mani Pokhrel, Keshab Parajuli, Shyam Kumar Mishra, Sangita Sharma, Niranjan Shah, Hari Prasad Kattel, Subhash Dhital, Sulochana Khatiwada, Narayan Parajuli, Manoj Pradhan, Basista Prasad Rijal

**Affiliations:** 1 HIV Reference Unit, National Public Health Laboratory, Kathmandu, Nepal; 2 Department of Microbiology, Tribhuvan University Teaching Hospital, Kathmandu, Nepal; 3 Department of Microbiology, Universal Medical College, Bhairahawa, Nepal; 4 Department of Microbiology, ManMohan Memorial Institute of Health Sciences, Kathmandu, Nepal; 5 Department of Microbiology, Nepalese Army Institute of Health Science, Kathmandu, Nepal

**Keywords:** *Candida*, CHROMagar, Antifungal susceptibility testing

## Abstract

**Background:**

*Candida* species are responsible for various clinical infections ranging from mucocutaneous infection to life threatening invasive diseases along with increased resistance to antifungal drugs has made a serious concern. Resistance to antifungal agents has increased during the last decade. Thus, identification of *Candida* up to species level and its antifungal susceptibility testing has a paramount significance in the management of Candidal infections. The aim of the study was to speciate *Candida* species and to determine antifungal susceptibility pattern of *Candida* species to antifungal agents.

**Methods:**

A total of 100 consecutive *Candida* species were isolated from 1248 clinical specimens over 7 months period. Growths on Sabouraud dextrose agar were evaluated for colony appearance, macroscopic examination, Gram staining, germ tube test and urea hydrolysis test. Further, they were processed for *Candida* speciation on CHROMagar. Antifungal susceptibility testing was performed as recommended by Clinical and Laboratory Standards Institute (CLSI) M44-A document.

**Results:**

Out of 100 *Candida* isolates, *Candida albicans* (56%) was the most common species. Among the non-*albicans Candida* species, *Candida tropicalis* (20%) was the predominant isolate followed by *Candida glabrata* (14%). Regarding antifungal susceptibility pattern, *Candida* species were more susceptible to clotrimazole (82%) followed by fluconazole (64%) and miconazole (44%).

**Conclusions:**

*Candida albicans* was the predominant species responsible for various Candidal infections. Among commonly used antifungal drugs clotrimazole, miconazole and fluconazole were most effective.

## Background

*Candida* species is a normal commensal flora of human body inhabiting skin, mucous membranes and gastrointestinal tract but may be associated with superficial and deep seated fungal infections [1]. The switch of *Candida* species from commensal to a potent pathogen is facilitated by various virulence factors such as adherence to host tissues, medical devices, biofilm formation, and secretion of extracellular hydrolytic enzymes [2]. Also, in recent year non-*albicans Candida* (NAC) species are considered as major pathogens causing severe infections in human [3].

The commonly used antifungal drugs show significant variation in the susceptibility pattern among the types of *Candida* species. The drug resistance scenario has been increasing during last decades due to over growing use of random antifungal agents [4]. Several previous studies reported the emergence of drug resistance *Candida* species in global scenario [5, 6]. Therefore, the change in drug susceptibility pattern of *Candida* species and introduction of newer antifungal agents has made the in vitro susceptibility testing of antifungal agents more relevant for using specific and sensitive drugs.

Thus, the isolation, identification, characterization and susceptibility testing of *Candida* species in clinical specimens have become increasingly important for management of fungal infections. CHROMagar medium is an easy, rapid and reliable method to isolation and for differentiation of four types of *Candida* species [7].

In the present study, we explored the characterization of *Candida* species using CHROMagar and showed the susceptibility pattern of *Candida* isolates from clinical specimens.

## Methods

A laboratory based cross sectional study was carried out in the Department of Microbiology, Tribhuvan University Teaching Hospital from July 2014 to January 2015. A total of 1248 different clinical specimens (urine, sputum, catheter tip, blood, high vaginal swabs and endotracheal tube) proceeded for laboratory investigation. The preliminary diagnoses of specimens were performed by wet mount, Gram stain, culture on Sabouraud dextrose agar (SDA) and urea hydrolysis test. The isolates diagnosed to be fungus other than *Candida* species were excepted from the study. For the clinical significance of *Candida* isolates from sputum and urine, the specimens were analysed by microscopy as well for the evidence of budding yeast cell with pseudohyphae along with significant pus cells [8, 9]. All samples were inoculated on Sabouraud dextrose agar (SDA) slants supplemented with chloramphenicol and aerobically incubated at 37 °C for 24–48 h. For blood culture, 8–10 ml venous blood was collected aseptically and cultured in 45 ml Brain heart infusion (BHI) broth. It was then incubated at 37 °C for up to 96 h before reported as no growth.

Any visible growth seen on SDA slope was processed for identification of the species. From an isolated colony, macroscopic examination, Gram staining, germ tube test and urea hydrolysis test was performed. The yeasty, pasty and creamy colony that showed Gram positive budding yeast cells with pseudohyphae on microscopic examination and negative urea hydrolysis test were further processed for *Candida* speciation on CHROMagar. *Candida* species were differentiated based on type of the growth and colour of isolates on CHROMagar *Candida* (HiMedia, Mumbai, India) [10, 11]. After incubation at 37 °C for 24–48 h, colour of colonies was observed on CHROMagar (*C. albicans*—light green, *C. glabrata*—cream to white, *Candida krusei*—purple, fuzzy and *C. tropicalis*—blue to purple).

Antifungal susceptibility testing was performed and interpreted for all the isolates of *Candida* using disc diffusion method as recommended by Clinical and Laboratory Standards Institute (CLSI) M44-A document guidelines [12]. The inoculum was prepared by suspending five colonies of growth in 5 ml of sterile saline and compared the turbidity to 0.5 McFarland Standard. A cotton swab was dipped into the inoculum suspension and evenly streaked onto Mueller–Hinton agar supplemented with 2% glucose and 5 µg/ml methylene blue [13, 14]. *C*. *albicans* ATCC 90028, *C. tropicalis* ATCC 750 were used as controls.

Antifungal discs containing fluconazole (25 µg), ketoconazole (15 µg), clotrimazole (10 µg), and miconazole (10 µg) were placed on the inoculated media. Zone of inhibition around the disc was measured after incubating the media at 37 °C for 24 h. In case of *C. krusei*, repeated antifungal susceptibility testing to fluconazole was performed [12, 15].

## Results

A total of 100 *Candida* species were isolated from urine (48%), sputum (42%), catheter tip (4%), blood (2%), high vaginal swabs (2%) and endotracheal tube (2%) as shown in Table 1. We analysed 1248 clinical specimens and that showed 8.1% culture positivity. Gender-wise distribution showed that 52% *Candida* isolates were from male and 48% from female. Based on CHROMagar, four types of *Candida* species were differentiated. *C. albicans* (56%) was the most frequently encountered species and the bulk of isolates were from urine and sputum samples (26 and 24% respectively). Among the NAC spp., *C. tropicalis* (20%) was the commonest isolate followed by *C. glabrata* (14%) and *C. krusei* (10%) respectively. All four types of *Candida* species were isolated from urine samples whereas *C. albicans* was isolated from all specimens except the endotracheal tube. The different species of *Candida* reported from various specimens in our study is showing in Table 1.

**Table 1:** Distribution frequency of *Candida* species obtained from various clinical specimens

Specimens	*Candida* spp.				Total
	*C. albicans*	*C. tropicalis*	*C. krusei*	*C. glabrata*	
Urine	26	12	2	8	48
Sputum	24	8	4	6	42
Catheter tip	2	0	2	0	4
Blood	2	0	0	0	2
High vaginal swab	2	0	0	0	2
Endotracheal tube	0	0	2	0	2
Total	56	20	10	14	100

Overall, antifungal susceptibility profile of *Candida* species to ketoconazole was found to be 6% susceptible (S), 8% susceptible dose dependent (SDD) and 86% resistant (R). In similar way, antifungal profile (S, SDD, R) to fluconazole was (64, 16 and 20% respectively), to miconazole (44, 44, 12%) and to clotrimazole (82, 12, 6%) as depicted in Table 2. Among the four antifungal agents, the highest level of susceptibility was observed in clotrimazole, followed by fluconazole and miconazole respectively whereas ketoconazole showed the highest level of resistance.

**Table 2:** Antifungal susceptibility testing of various *Candida* spp.

Antifungal agents	*Candida* spp.				Total (N = 100)
	*C. albicans* (N = 56)	*C. tropicalis* (N = 20)	*C. krusei* (N = 10)	*C. glabrata* (N = 14)	
Ketoconazole					
S	4 (7.2%)	0 (0%)	2 (20%)	0 (0%)	6 (6%)
SDD	2 (3.6%)	4 (20%)	0 (0%)	2 (14.3%)	8 (8%)
R	50 (89.3%)	16 (80%)	8 (80%)	12 (85.8%)	86 (86%)
Fluconazole					
S	40 (71.5%)	12 (60%)	4 (40%)	8 (57.2%)	64 (64%)
SDD	6 (10.8%)	4 (20%)	6 (60%)	0 (0%)	16 (16%)
R	10 (17.9%)	4 (20%)	0 (0%)	6 (42.9%)	20 (20%)
Miconazole					
S	30 (53.6%)	10 (50%)	2 (20%)	2 (14.2%)	44 (44%)
SDD	18 (32.2%)	8 (40%)	6 (60%)	12 (85.8%)	44 (44%)
R	8 (14.3%)	2 (10%)	2 (20%)	0 (0%)	12 (12%)
Clotrimazole					
S	44 (78.6%)	16 (80%)	10 (100%)	12 (85.8%)	82 (82%)
SDD	8 (14.3%)	4 (20%)	0 (0%)	0 (0%)	16 (16%)
R	4 (7.2%)	0 (0%)	0 (0%)	2 (14.2%)	6 (6%)

In this study, *C. albicans* (89.3%) was found more resistant to ketoconazole with compared to NAC spp. Among the NAC spp., *C. krusei* showed 20% sensitivity whereas no sensitive results were observed in *C. tropicalis* and *C. glabrata*. In fluconazole, more resistance was observed in *C. glabrata* (42.9%), whereas no resistance was observed for *C. krusei*. *C. albicans* isolates (71.5%) were more sensitive to fluconazole with compared to NAC spp.

In miconazole, more resistance was observed for *C. krusei* (20%) while no resistance was observed for *C. glabrata*. *C. albicans* isolates (53.6%) were more sensitive to miconazole compared to NAC spp. In clotrimazole, more resistance was observed in *C. glabrata* (14.2%), no resistance was observed in *C. tropicalis* and *C. krusei*. All isolates of *C. krusei* were found to be sensitive to clotrimazole. Antifungal susceptibility profile of different *Candida* species to antifungal agents is showing in Table 2.

## Discussion

In this study, a total of 100 *Candida* isolates were obtained over 7 months of period from different clinical specimens. The majority of *Candida* species were isolated from urine and sputum that cover 90%, which indicates the higher incidence and distribution of *Candida* species causing urinary tract and respiratory tract infections.

Of the 100 *Candida* isolates, *C. albicans* was the predominant species (56%) followed by *C. tropicalis* (20%), *C. glabrata* (14%) and *C. krusei* (10%) respectively. Our finding shows similar prevalence scenario of *Candida* species and susceptibility pattern to the previous data reported by two independent groups from India which showed *C. albicans* is more prevalent among the *Candida* isolates [16, 17]. Similar study conducted by Sajjan et al. also reported *C. albicans* as the major isolate. Among the NAC species, *C. tropicalis* was most prevalent followed by *C. glabrata* and *C. krusei* respectively [18]. Jayalakshmi et al. also showed that *C. tropicalis* (26.6%) was prevalent among the NAC species [19]. Similar result has been depicted in various studies conducted in different countries of Europe [20–22]. However, many studies have shown that NAC species have more isolation rate than *C. albicans* which suggest the emergence of non-*albicans Candida* species as important pathogens [23, 24].

Speciation of *Candida* species by CHROMagar on the basis of colour differentiation offered a rapid, convenient and reliable method for identification of clinically important *Candida* species when compared with cumbersome traditional techniques. In developing countries, CHROMagar can be taken as a simple phenotypic test alternative to molecular based assay. CHROMagar has high sensitivity as well as specificity for the identification of *Candida* species [10, 25]. According to various finding from our regions, these four species are more prevalent, so we chose this medium for isolation of *Candida* spp. [17, 26]. It facilitates the detection and identification of *Candida* species from mixed culture and provides results within 24–48 h.

In this study, *Candida* species were found to be more susceptible to clotrimazole (82%) followed by fluconazole (64%) and miconazole (44%) respectively whereas 86% of the isolates were resistant to ketoconazole. Among the four antifungal agents, clotrimazole and miconazole had not been used for clinical treatment but might be of interest for mycological study. A similar study was conducted to perform an antifungal test to clotrimazole and miconazole as of mycological interest [27, 28]. In the present study, 20% of total isolates were found to be resistant to fluconazole by disc diffusion method. The highest rate of fluconazole resistance was observed in *C. glabrata* (42.9%), followed by *C. tropicalis* (20%) and *C. albicans* (17.9%). Our finding is very close with the findings of Mondal et al., which also showed 18% *Candida* spp. and 19.2% *C. tropicalis* were resistance to fluconazole. On the contrary, all *C. krusei* isolates were sensitive to fluconazole in our study while 60% were found to be susceptible dose dependent for the same. In a case of *C. krusei*, some degree of susceptibility was seen to fluconazole, comparable to other studies by applying same interpretative criteria as introduced by CLSI [15, 29]. However, it is reported as intrinsically resistant to fluconazole. So, it showed further investigation is needed for solving the query of fluconazole sensitivity. The increase in resistance to fluconazole is of serious concern as it is the most commonly used azole for superficial as well as deep candidiasis.

This study also reveals the higher resistant rate for ketoconazole (86%). This high level of resistance of ketoconazole might be due to overuse of antifungal agents and also their empirical therapy in our scenario. The higher rate of ketoconazole resistance was seen in *C. albicans* (89.3%), *C. glabrata* (85.8%), and followed by *C. tropicalis* and *C. krusei* (80% for both). Our result hugely differs from the findings by Binesh et al. in which only 2.1% *C. albicans* isolates were resistant to ketoconazole [30]. Furthermore, Mondal et al. revealed overall 11.7% resistance to ketoconazole and with *Candida krusei* (20%) followed by *C. glabrata* (17.6%), *C. tropicalis* (15.2%) and least being *C. albicans* (7.8%). The study conducted by Zomorodian et al. showed that fluconazole sensitivity was seen in 96.6% of the *Candida* isolates [31].

These findings suggest the rapid increase in resistance among *Candida* species for ketoconazole and need for speciation and antifungal susceptibility before treatment with antifungal drug.

## Conclusions

In our study we found that *C. albicans* was the predominant species responsible for various Candidal infections. Among commonly used antifungal drugs clotrimazole, miconazole and fluconazole demonstrated a high rate of sensitivities while ketoconazole was the least effective for both *C*. *albicans* and NAC spp. Species identification of *Candida* has a paramount effect on successful treatment as it helps in optimum selection of the therapeutic agent and use of CHROMagar is a simple, rapid and inexpensive method for identification of *Candida* species especially in the laboratory with limited resources.

## Abbreviations

CLSI: Clinical and Laboratory Standards Institute
SDA: sabouraud dextrose agar
NAC spp.: non-*albicans Candida* species
S: susceptible
SDD: suceptible dose dependent
R: resistant
*C. albicans*: *Candida albicans*
